# Microelectrode Array With Transparent ALD TiN Electrodes

**DOI:** 10.3389/fnins.2019.00226

**Published:** 2019-03-22

**Authors:** Tomi Ryynänen, Anssi Pelkonen, Kestutis Grigoras, Oili M. E. Ylivaara, Tanja Hyvärinen, Jouni Ahopelto, Mika Prunnila, Susanna Narkilahti, Jukka Lekkala

**Affiliations:** ^1^Micro- and Nanosystems Research Group, BioMediTech Institute and Faculty of Medicine and Health Technology, Tampere University, Tampere, Finland; ^2^NeuroGroup, BioMediTech Institute and Faculty of Medicine and Health Technology, Tampere University, Tampere, Finland; ^3^VTT Technical Research Centre of Finland Ltd., Espoo, Finland

**Keywords:** MEA, ALD, TiN, neurons, transparent, microelectrode

## Abstract

Low noise platinum black or sputtered titanium nitride (TiN) microelectrodes are typically used for recording electrical activity of neuronal or cardiac cell cultures. Opaque electrodes and tracks, however, hinder the visibility of the cells when imaged with inverted microscope, which is the standard method of imaging cells plated on microelectrode array (MEA). Even though transparent indium tin oxide (ITO) electrodes exist, they cannot compete in impedance and noise performance with above-mentioned opaque counterparts. In this work, we propose atomic layer deposition (ALD) as the method to deposit TiN electrodes and tracks which are thin enough (25–65 nm) to be transparent (transmission ∼18–45%), but still benefit from the columnar structure of TiN, which is the key element to decrease noise and impedance of the electrodes. For ALD TiN electrodes (diameter 30 μm) impedances from 510 to 590 kΩ were measured at 1 kHz, which is less than the impedance of bare ITO electrodes. Human induced pluripotent stem cell (hiPSC)-derived cortical neurons were cultured on the ALD TiN MEAs for 14 days without observing any biocompatibility issues, and spontaneous electrical activity of the neurons was recorded successfully. The results show that transparent ALD TiN film is a suitable electrode material for producing functional MEAs.

## Introduction

Possibly the simplest and therefore very popular method to perform *in vitro* electrophysiological measurements for neuronal and cardiac cells is to plate the cells on a microelectrode array (MEA). Common MEA applications vary from basic biological research and *in vitro* disease modeling to drug screening and toxicology studies. The standard commercial MEA typically contains 60 electrodes in 8 × 8 and the electrode diameter is usually 10–50 μm. Such small electrodes have rather high impedance and noise levels due to small contact area with the cells and culturing medium. In order to decrease noise and impedance, the active surface area of the electrode can be increased by using columnar or porous electrode materials like titanium nitride (TiN) (Egert et al., 1998; Ryynänen et al., 2018b) or platinum black (PtB) (Thomas et al., 1972; Pine, 1980; Oka et al., 1999). However, in addition to electrical measurements, it is often necessary to image and observe the cells growing or fixed on the MEA. Because imaging from the topside through the cell culturing medium has several optical challenges, inverted microscopes are favored in cell studies. With MEAs this leads to another challenge – the opaque electrodes and tracks lie now between the cells and the microscope optics, i.e., the cells or cellular processes located on the electrodes, which are often the most interesting points to observe, are not visible. To overcome this challenge MEAs with transparent indium tin oxide (ITO) electrodes and tracks have been used occasionally (Nam et al., 2006; Tang et al., 2006; Kim et al., 2013; Qwane Biosciences, 2016). For tracks, ITO is a valid choice, but as a non-porous material, both the impedance and noise level of the ITO electrodes are relatively high, comparable to planar gold electrodes. Thus, ITO electrodes are usually coated with TiN or PtB in order to achieve tens of times or even larger decrease in the impedance of the electrodes compared with pure ITO electrodes (Jimbo, 2007; Qwane Biosciences, 2016). At the same time the visibility through the electrodes is, however, lost.

There have been some efforts to fabricate transparent graphene (Du et al., 2015) or diamond electrodes (Granado et al., 2015), but they both require rather laborious fabrication processes and special expertise, and at least so far their performance has not been notably better than that of ITO electrodes. Instead of introducing a new transparent electrode material, in this work, we have taken a benefit of the fact that basically whatever material is transparent if the layer is just thin enough. However, usually this means that the film thickness is at maximum only a few tens of nanometers and at that low thicknesses it is not at all self-evident whether the film is fully continuous and homogenous. In sputtering (Egert et al., 1998) and ion beam assisted e-beam deposition (IBAD) (Ryynänen et al., 2018b) which so far have been used for depositing TiN electrodes, the thin film formation is somewhat flocculated. On the contrary, in atomic layer deposition (ALD), where the precursors are dosed one-by-one and self-terminating surface reactions take place, films are grown on atomic or at least molecular layer-by-layer manner, the film can be expected to be uniform already at sub nanometer thicknesses (Sintonen et al., 2014). The porous or columnar like structure of TiN is, however, present already at very thin ALD TiN films. In this work, we propose using ALD TiN electrodes as potential solution to fabricate transparent MEAs with lower impedance compared with ITO electrodes.

## Materials and Methods

### MEA Fabrication and Characterization

The ALD TiN MEA fabrication was started by growing ALD TiN on 49 mm × 49 mm × 1 mm microscope slide grade soda lime glass substrates (Gerhard Menzel GmbH) using SUNALE R-150B reactor (Picosun). The ALD process temperature was 450°C and TiCl_4_ and ammonia were used as precursors and nitrogen as a carrier gas with pulse/purge time 0.2 s / 2 s and 2 s / 4 s for TiCl_4_ and ammonia, respectively. The process is described in more detail in Grigoras et al. (2016). Three different film thicknesses were obtained by repeating 1000, 2000, and 3000 ALD cycles, respectively. At the same time reference samples were grown on silicon wafers coated with silicon dioxide. Another batch of glass substrates and Si wafers was coated for characterization purposes. Samples were imaged with scanning electron microscope (SEM) LEO1560 (Zeiss) to visualize the morphology and for the thickness measurements. In addition, surface RMS (root mean square) roughness was measured by AFM (Digital Instruments DI3100, tapping mode using silicon probe Tap150Al-G with radius <10 nm, scan rate 1 Hz). Sheet resistance of the thin films was measured using four-point probe Loresta AP MCP-T 400 (Mitsubishi Co.). Fifteen measurements on glass and four measurements on silicon, for each thickness were averaged. Transparency of the ALD TiN films deposited on glass substrates that were later processed as MEAs were measured with Ocean Optics JAZ spectrometer over wavelength range from 350 to 1000 nm.

Unlike in standard 8 × 8 layout by Multi Channel Systems MCS GmbH which has 59 30 μm electrodes and one grounding electrode, our design contains only 56 30 μm electrodes as we have two grounding electrodes and two other bigger electrodes for process characterization. The MEA fabrication was continued by wet etching 8 × 8 electrode layout (electrode diameter 30 μm and pitch 200 μm), tracks, and contact pads on the ALD TiN layer. At first an HF:H_2_O_2_:H_2_O mixture was applied to remove the oxide and after that the rest of the etching was performed with 30% H_2_O_2_ at 50°C with etching times of 2.5, 3.5, and 4.5 min, respectively, for different TiN thicknesses. Next, an insulator layer consisting of 100 + 500 + 100 nm stack of SiO_2_:SiN:SiO_2_ was deposited by plasma enhanced chemical vapor deposition (PECVD) over the TiN layer. Openings for the electrodes and the contact pads were dry etched with SF_6_ + O_2_ RIE process (Williams et al., 2003). Two MEAs of each ALD TiN thicknesses were fabricated, and for another of each thickness an additional 400 nm of TiN coating was deposited on the contact pads and large ground electrodes with IBAD technique (Ryynänen et al., 2018b) to enhance the mechanical durability.

PDMS rings were reversibly bonded to ready-made MEAs and about 1 h after filling the ring with DPBS (PBS Dulbecco w/o Ca++, Mg2+, Biochrom GmbH) impedances of the electrodes were measured with MEA-IT (Multi Channel Systems MCS GmbH) impedance tester at 1 kHz. At first, an average of two measurements was calculated for each electrode as the impedance value. Next, electrodes whose impedance was not within ±0.25 times the median of all electrodes of the same MEA were excluded as faulty electrodes. The included electrodes were used to calculate mean and standard deviation (SD) for that MEA.

### Cell Experiments

In order to verify ALD TiN electrodes’ biocompatibility, durability and performance in real cell experiments, they were cultured with human induced pluripotent stem cell (hiPSC)-derived cortical neurons (cell line 10212.EURCCs). BioMediTech has a supportive statement from the regional ethical committee of Pirkanmaa Hospital District for derivation, culturing, and differentiation hiPSCs (R08070). The hiPSCs were expanded in feeder-free culture as previously described (Hongisto et al., 2017). The neuronal cell differentiation and maintenance mediums are based on those described earlier (Shi et al., 2012) with further in-house modifications. Cell culture plates were coated with poly-L-ornithine (PO, Sigma) and LN-521 (BioLamina). Cells were cultured in neural maintenance medium that consisted of 1:1 D-MEM/F12 with Glutamax and Neurobasal, 0.5% N2, 1% B27 with Retinoic Acid, 0.5 mM GlutaMAX, 0.5% NEEA, 50 μM 2-mercaptoethanol (all from Thermo Fisher Scientific), 2.5 μg/ml Insulin (Sigma) and 0.1% penicillin/streptomycin (Thermo Fisher Scientific). For the first 12 days cells were cultured in neural maintenance medium supplemented with 100 nM LDN193189 and 10 μM SB431542 (both from Sigma). After that neural progenitor cells were expanded in neural maintenance medium supplemented with 20 ng/ml fibroblast growth factor-2 (FGF2) (R&D Systems). From day 26 onward, the neural maintenance medium was supplemented with 20 ng/ml brain-derived neurotrophic factor (BDNF, R&D Systems), 10 mg/ml glial-derived neurotrophic factor (GDNF, R&D Systems), 500 μM dibutyryl-cyclicAMP (db-cAMP, Sigma) and 200 μM ascorbic acid (AA, Sigma).

The MEAs were coated for cell plating with 0.1% polyethylenimine (PEI; Sigma-Aldrich) in borate buffer (1 h RT; excess washed away 3× with sterile H_2_O) and with 50 μg/ml human LN-521 (1 h, +37°C). Cells were plated in density of 10^6^/cm^2^. All medium was always changed on the day before MEA measurements, and altogether four times per week. The hiPSC-derived cortical neurons were cultured for 14 days on the ALD TiN MEAs, and for control commercial MEA with opaque sputtered TiN electrodes (MCS 60-6wellMEA200/30iR-Ti-w/o; electrode diameter and pitch identical to the ALD TiN MEAs). MCS MEA2100 system was used to record spontaneous activity of the cells on 3, 7, 10, and 14 days on MEAs. Measurements were done with sampling rate of 25 kHz. The amplifier’s high-pass cutoff frequency was 1 Hz and low-pass 3 kHz. Thereafter, the signal was digitally filtered in MC_Rack software (MCS) using a 2nd order Butterworth high-pass filter with the cutoff of 200 Hz. Neuronal spikes were detected from the filtered data when their amplitude exceeded the threshold of -5 × SD of noise. Together with the MEA measurements, the MEAs were imaged using an Axio Observer.A1 inverted microscope equipped with an Axiocam 506 color camera (both from ZEISS).

At the end of the cell experiment, the cells on selected MEAs were fixed with 4% paraformaldehyde and stained with method adapted from Lappalainen et al. (2010) for nuclei (4^′^,6-diamidino-2-phenylindole [DAPI]), and neuronal markers β-tubulin_III_ (BTUB3, 1:500; T8660, Sigma-Aldrich) and neurofilament heavy chain (NF-H, 1:250; A00136, GenScript). Alexa Fluor 488 (1:400; A21202, Thermo Fisher) and Alexa Fluor 568 (1:400; A11041, Thermo Fisher) were used for secondary antibody labeling. The maximal excitation and emission wavelengths were 358 and 461 nm for DAPI, 495 and 519 nm for Alexa Fluor 488, and 578 and 603 nm for Alexa Fluor 568 (respectively). Fluorescence imaging was done using an IX51 microscope equipped with a DP30BW camera (both from Olympus Corporation).

## Results

**FIGURE 1:**
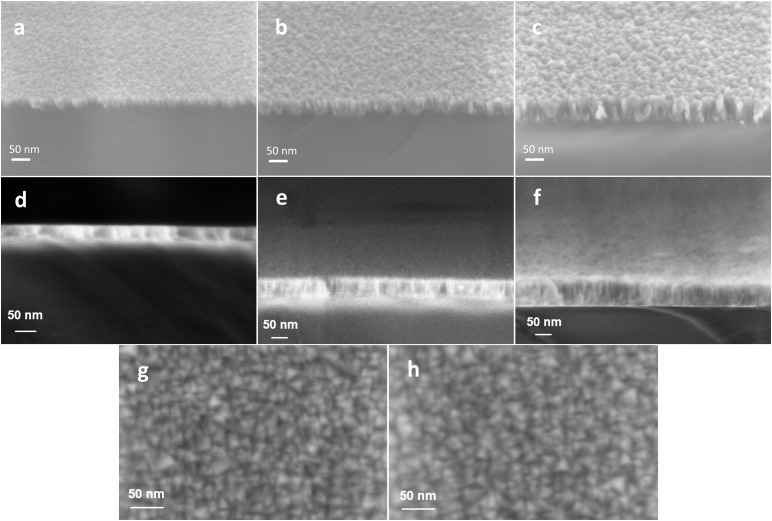
**(a–c)** SEM images with 30° tilted view reveal columnar structure of ALD TiN thin films deposited on Si reference substrates. **(d–f)** SEM images from corresponding ALD TiN films deposited on glass substrates. Number of ALD cycles: **(a,d)** 1000, **(b,e)** 2000, and **(c,f)** 3000. Practically identical crystal size in SEM top views of 3000 cycles deposited on **(g)** Si and **(h)** glass substrate.

**FIGURE 2:**
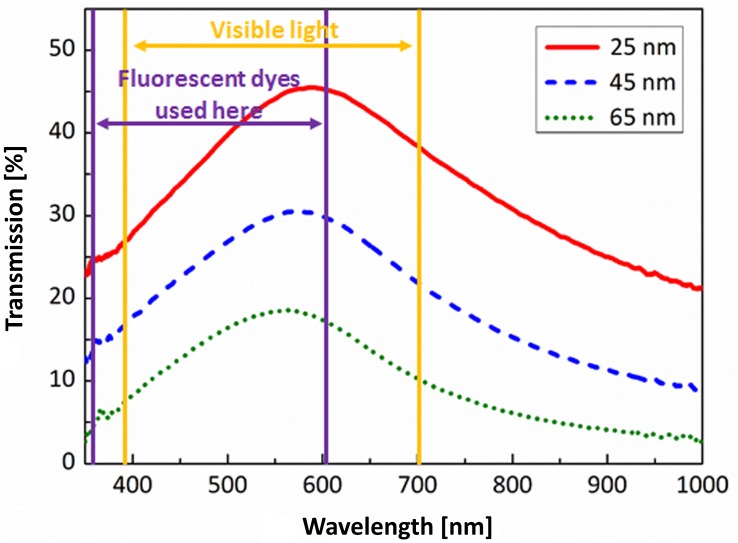
Transmission of ALD TiN thin films as a function of wavelength. The spectrum of visible light ranges approximately from 390 to 700 nm. The fluorescent dyes used in this work have excitation and emission peaks ranging from 358 to 603 nm. However, there are dyes that have these peaks as high as 800 nm, at the near-infrared range.

The ALD cycles 1000, 2000, and 3000 led to approximately 25, 45, and 65 nm, respectively, TiN film thicknesses on glass substrate. SEM images taken from the reference samples grown on Si (Figure 1a–c) reveal that ALD TiN films had a highly columnar morphology already at the lowest thickness and the column/grain size got bigger when the thickness was increased. SEM imaging of samples grown on glass substrate (Figure 1d–f), on the contrary, suffered from significant charging effect and we were unable to get fully comparable images to evaluate the column size difference between films grown on Si and glass. However, the SEM images taken from the top side (Figure 1g,h) show that on films grown on both substrates the surface structure is practically equal, containing 10–25 nm size grains. The AFM measurements (Supplementary Figure S1) confirm that the RMS roughness increases with the film thickness for both substrates. For glass substrate without TiN layer the RMS roughness was 0.40 nm and for Si 0.22 nm. Table 1 summarizes the layer thicknesses and RMS roughness values. The table includes also sheet resistances which decrease with thickness as wells as calculated resistivity values. The transparency of the ALD TiN electrodes at different wavelengths of light is shown in the complete transmission curves in Figure 2. Transmission was maximal in all three layer thicknesses at approximately 575 nm and the values can be found also from Table 1.

**Table 1:** Layer thickness, RMS roughness, sheet resistance, resistivity, and transmission (@ 575 nm) for 1000, 2000, and 3000 ALD cycles of ALD TiN grown on glass and silicon substrates.

	Glass			Silicon		
ALD cycles	1000	2000	3000	1000	2000	3000
Thickness [nm]	25 ± 5	45 ± 5	65 ± 3	21 ± 3	41 ± 3	62 ± 3
RMS roughness [nm]	1.08 ± 0.05	1.75 ± 0.05	2.20 ± 0.05	0.98 ± 0.04	1.69 ± 0.04	1.98 ± 0.02
Sheet resistance [Ω/sq]	199 ± 25	70 ± 8	47 ± 5	128 ± 8	55 ± 5	41 ± 2
Resistivity [μΩ-cm]	498 ± 60	315 ± 33	305 ± 33	269 ± 25	226 ± 20	252 ± 12
Transmission [%]	45 ± 5	30 ± 5	19 ± 5	–	–	–


**FIGURE 3:**
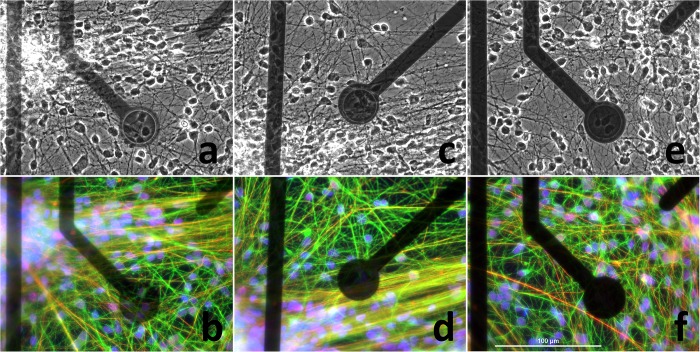
Neuronal cells on ALD TiN MEAs imaged with an inverted microscope and 40× magnification. On the top row there are bright field images and on the bottom row fluorescence images. ALD TiN thickness are **(a,b)** 25 nm, **(c,d)** 45 nm, and **(e,f)** 65 nm. In the immunofluorescence images nuclei (DAPI) are shown in blue, BTUB3 in green and NF-H in red. The maximal excitation and emission wavelengths are 358 and 461 nm for the nuclear dye, 495 and 519 nm for the BTUB3 dye, and 578 and 603 nm for the NF-H dye (respectively). Scale bar (100 μm) for the images is shown in **(f)**.

**FIGURE 4:**
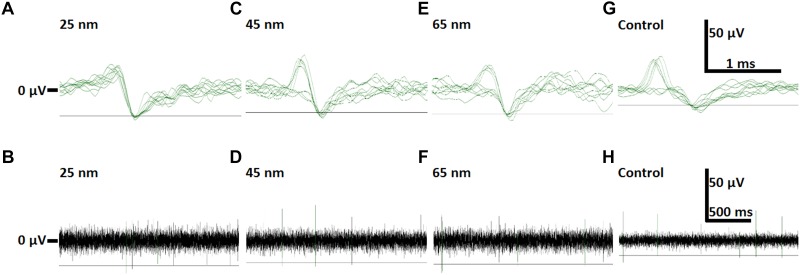
Examples of neuronal activity detected with transparent ALD TiN electrodes of different thicknesses and commercial opaque TiN MEA (control). All data is high-pass filtered above 200 Hz. The gray horizontal line in each subfigure represents the signal detection threshold (–5 × SD of noise). On the top row there are overlays of 10 spikes from **(A)** 25 nm, **(C)** 45 nm and **(E)** 65 nm thick ALD TiN electrodes, as well as a commercial control MEA **(G)**. The displayed traces were recorded 3 **(C)**, 7 **(A)** and 14 **(E,G)** days after plating on MEA. The electrodes were capable of detecting signals from multiple cellular sources, which is shown here as both mono- and biphasic waveforms detected by the same electrodes. The bottom row **(B,D,F,H)** shows corresponding 2 s traces from the same electrodes. The 0 μV level is indicated for the top row in **(A)** and for the bottom row in **(B)**. The voltage and time scale bars for the top row are shown in **(G)** and for the bottom row in **(H)**.

The impedance values of ALD TiN MEAs and for comparison also datasheet values of commercial TiN and ITO MEAs are presented in Table 2. In the two thinnest versions of MEAs only 0–2 electrodes were faulty in each. Usually meaning some fatal scratch or dirt across the track or electrode or just temporary contact problem in MEA-IT. On the contrary, in the thickest MEAs there were 11 or 15 electrodes which were basically working but were excluded from the impedance calculations because of not filling the impedance within ±0.25 times the median criteria. In both MEAs those electrodes were in the same quadrant, which indicates some one-time unknown localized inhomogeneity or human error either in some deposition (ALD or PECVD) or etching step, or in photolithography, and which we do not expect to re-appear in the future batches. The neuronal cells were visible through the electrodes and the tracks of all three ALD TiN thicknesses in bright field fluorescence images. In fluorescence staining, 65 nm thickness of ALD TiN blocked the signal visibility, whereas the thinner ALD TiN layers allowed visualization of staining (Figure 3). Despite the higher impedance compared with commercial opaque TiN electrodes, all the ALD TiN MEAs recorded biological signals comparable to commercial MEAs (Figure 4). An overview of a typical recording with all electrodes is shown in Supplementary Figure S2. Like the commercial control MEAs, also the ALD TiN MEAs detected both mono- and biphasic signals. No biocompatibility issues concerning ALD TiN were observed during the cell experiments.

**Table 2:** Measured impedances (mean ± SD @ 1 kHz) of ALD TiN MEAs with three different TiN thickness and with and without IBAD TiN strengthened contact pads.

Electrode material	ALD TiN thickness [nm]	IBAD TiN contact pads	Impedance [kΩ]	Number of valid electrodes (max 56)
ALD TiN (1000 cycles)	25 nm	yes	590 ± 50	55
		no	830 ± 80	55
ALD TiN (2000 cycles)	45 nm	yes	550 ± 50	56
		no	640 ± 40	54
ALD TiN (3000 cycles)	65 nm	yes	1060 ± 100^∗^	45
		no	510 ± 40	41
Opaque TiN (MCS)			30–100	
Transparent TiN (MCS)			<200	
ITO (Qwane)			900–1200	


*In addition, datasheet values of impedances (@ 1 kHz) of some commercial MEAs are given for comparison. Electrode diameter in all the MEAs is 30 μm, except 50 μm in Qwane’s ITO MEAs. ^∗^Most likely there was some failure in MEA processing and the result may not be reliable.*

## Discussion

In this study, three different thicknesses (25, 45, and 65 nm on glass) of ALD TiN films were grown and their applicability as sole conducting material for transparent MEAs was evaluated. The layer thicknesses of the films grown on Si were 5–10% lower than on glass. However, because of charging effect the thicknesses measured from the glass samples are less precise. The highly columnar morphology of ALD TiN seen in SEM images resembles the morphology that has been reported earlier for sputtered TiN electrodes (Egert et al., 1998). As expected, the transmission decreased linearly as a function of the film thickness and also the sheet resistance decreased as the film thickness was increased. Calculated resistivity of TiN was lower for layers deposited on silicon surface, what can be caused by lower initial roughness of silicon surface compared to glass, and different initial conditions on the surface: glass surface at 450°C could prevent formation of conducting TiN layer – what, most probably, causes much higher resistivity of the thinnest TiN layer on glass. In the case of silicon samples, the reason for some lower resistivity of layer obtained after 2000 cycles is not very clear. Anyway, within the accuracy limits, the value is close to numbers for other thicknesses.

Both increasing the film thickness and coating the contact pads with additional IBAD TiN layer for improved mechanical stability decreased the impedance. Compared with the sheet resistance, the change in ALD TiN thickness had moderate effect on the impedance. The lower impedance of thicker TiN layers may be due to the thicker tracks and the increased effective surface area by larger grain size. In future studies, the effect of tracks can be easily verified, e.g., by using the same track thickness and preferably smooth track material for each electrode thickness. It could be also worth to try ALD TiN with opaque Ti or Au tracks as an alternative for sputtered or IBAD TiN; whether already few tens on nm TiN layer would be sufficient to decrease the impedance to competitive level or is few hundreds of nm thick, and thus opaque layer needed to get the impedance below 100 kΩ. With the opaque thickness, ALD would otherwise be a valid alternative for those two deposition methods, but the low deposition rate makes it less competitive at high thicknesses. Already depositing the transparent thicknesses takes hours, e.g., about 2 h for 1000 cycles or 25 nm. However, considering the total MEA fabrication time the TiN deposition time is not necessarily so critical factor, especially if the reactor size allows deposition of the full batch in one run. Optimizing the pulse and purge times may also make it possible to shorten the deposition time remarkably in the future.

The effect by the IBAD contact pads on the impedance was clearer. Especially with the thinnest version, strengthening the contact pads seems justified to get the best contact and thus lower impedance (590 kΩ vs. 830 kΩ). Without IBAD TiN layer, it is obvious that the non-optimal contact contributes to the total impedance in addition to the electrode. This is a good reminder of the importance of not only paying attention to the properties of the electrodes, but also securing a good contact to the measurement electronics is important for the best MEA performance. The MEA version having 65 nm ALD TiN electrodes and IBAD TiN contact pads did not follow the trend of IBAD TiN coating giving lower impedance, and most likely, there has been some major failure in the fabrication of that MEA which explains the high impedance over 1060 kΩ vs. 510 kΩ without coating. For example, etching the openings for the electrodes to the insulator layer may have been incomplete or the etching time may have been too long leading to thinned electrode. Without this failure and earlier mentioned localized error affecting one quadrant of both 65 nm MEAs, otherwise the yield was very high and relatively small SD in impedances (∼10%) in all MEA versions indicates also excellent reproducibility.

If the impedance of our MEAs is compared with MEAs having transparent ITO electrodes and ITO tracks, the datasheet from a former MEA manufacturer, Qwane Biosciences (2016), gives impedance 900–1200 kΩ (electrode diameter 50 μm) for the only known commercial MEAs having bare ITO electrodes. Among the surprisingly small number of reports dealing with bare ITO electrodes Kim et al. (2013) show much higher noise level in bare ITO electrodes compared with ones having Au nanoparticle coating. Unfortunately, together with Tang et al. (2006) they do not mention the impedances of their ITO electrodes at all, whereas Nam et al. (2006) report as small as 535 kΩ impedance for their ITO electrodes. However, their result was presented in a preliminary study suggesting highly unconventional PDMS insulator layer and no exact details of the MEA structure and layout used in the impedance measurement were given. In any case, it is relatively safe to state that our ALD TiN electrodes have lower impedance than bare ITO electrodes of the same size would have.

Mierzejewski et al. (2018) have taken benefit of the same thin TiN idea as we do and have recently reported a transparent MEA with thin sputtered TiN electrodes and ITO tracks. They report an increase of impedance of approximately 150 kΩ compared with opaque TiN electrodes. MCS commercialized Mierzejewski’s MEA and the datasheet (60tMEA200/30iR-ITO) states impedance to be <200 kΩ One factor to explain the lower impedance compared with our electrodes could be the ITO tracks, which probably have been thicker and better conducting than our very thin TiN tracks. Moreover, of course, there undoubtedly are some differences in the properties between sputtered TiN and ALD TiN as well as in the maturity of the rest of the fabrication processes. Although ITO tracks may have certain advantages, the benefit of our approach is the simplicity of the structure, as just one deposition of conducting materials is needed.

In summary, very thin ALD TiN film was proposed to be utilized as the electrode and track material for transparent MEAs. 25 and 45 nm thick electrodes were found transparent in both bright field and fluorescence imaging, whereas 65 nm electrodes blocked fluorescence signal. The impedances of thin ALD TiN microelectrodes were lower than the impedance of transparent ITO electrodes in the literature. Despite about 10–15 times higher impedance compared with commercial opaque TiN electrodes all the ALD TiN MEAs recorded biological signals comparable to ones recorded with commercial MEAs. During the cell experiments no biocompatibility nor mechanical stability issues concerning ALD TiN were observed, except for the contact pads an additional IBAD TiN coating was found to be useful. We conclude that very thin ALD TiN film is a suitable material for producing transparent microelectrodes capable of measuring neuronal activity *in vitro*.

## Data Availability

The datasets generated for this study are available on request to the corresponding author.

## Author Contributions

TR was responsible for the MEA fabrication and characterization, and wrote the manuscript with the support from other authors. KG and OY were responsible for the ALD thin film depositions and characterization. TH produced the cells for experiments. AP was otherwise responsible for the cell experiments. JA, MP, SN, and JL provided additional support and participated in designing the experiments.

## Conflict of Interest Statement

The authors declare that the research was conducted in the absence of any commercial or financial relationships that could be construed as a potential conflict of interest.
