# Supplementary material for: Microelectrode Array With Transparent ALD TiN Electrodes
Source: Front Neurosci. 2019 Mar 22;13:226. doi: 10.3389/fnins.2019.00226 (PMC6438859; doi:10.3389/fnins.2019.00226)
Supplement: Supplementary file 1 [file Data_Sheet_1.pdf]

Supplementary Material

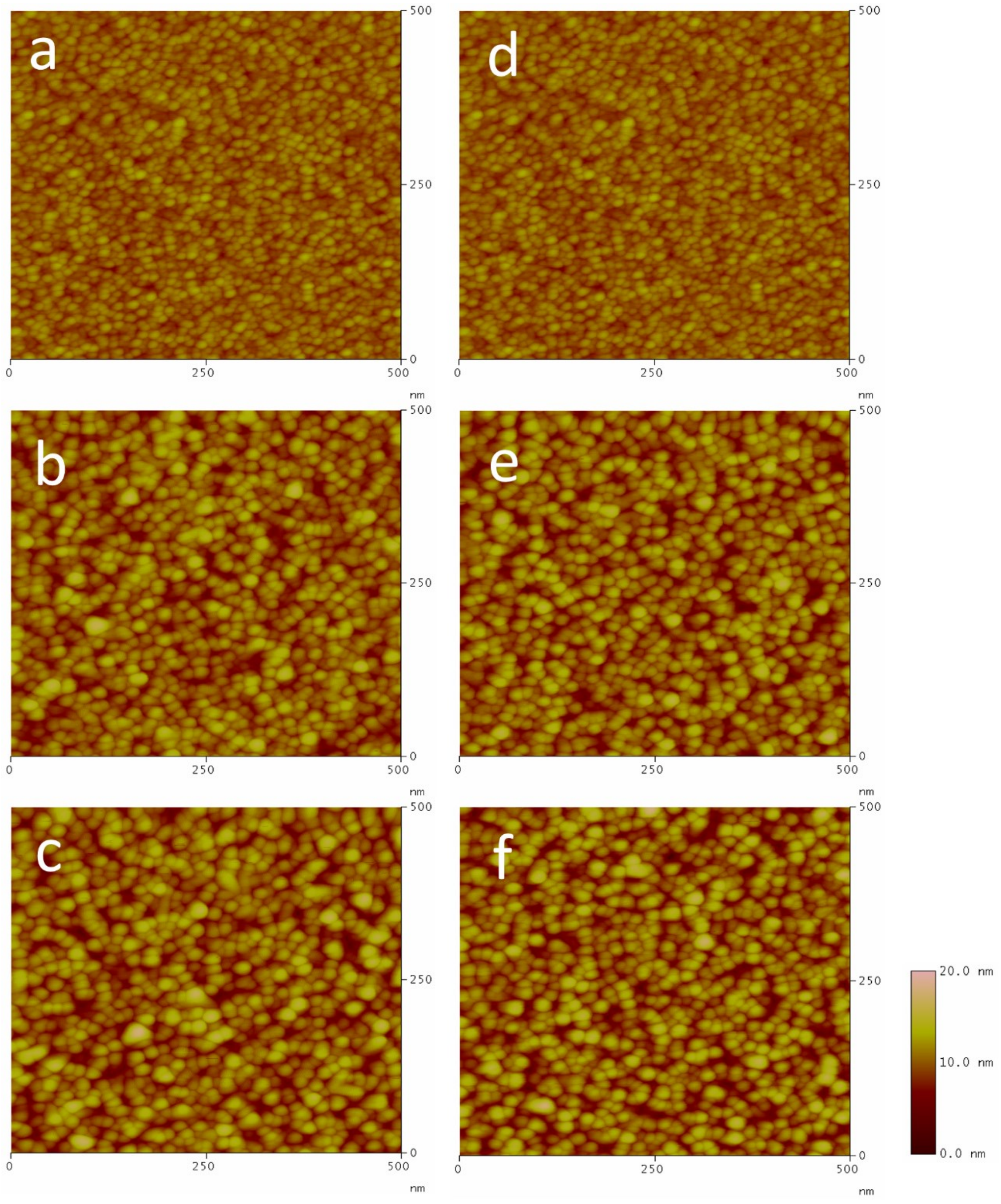

Supplementary Figure S1 | AFM images of ALD TiN surfaces on **(a-c)** silicon and **(d-f)** glass substrates. Number of ALD cycles: **(a,d)** 1000, **(b,e)** 2000, and **(c,f)** 3000.

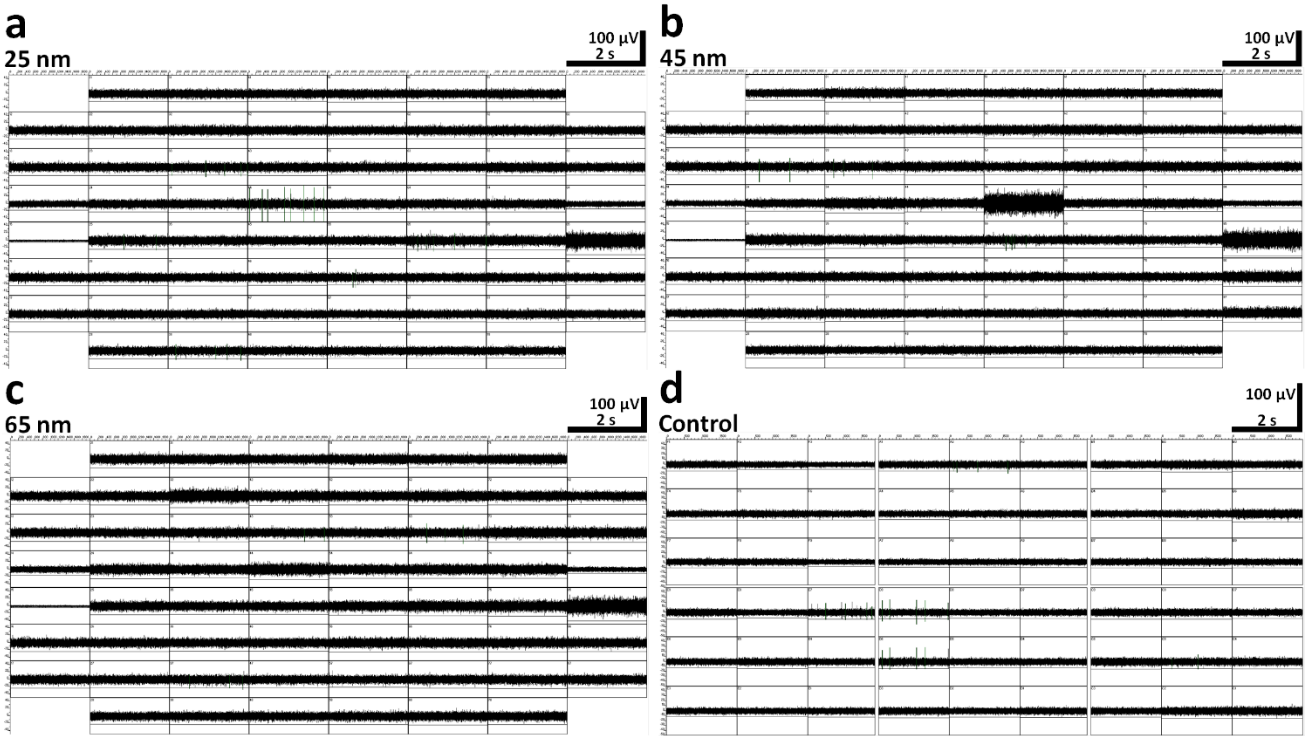

Supplementary Figure S2 | Overview of a typical recording of spontaneous activity on all electrodes of a device. All data was high-pass filtered above 200 Hz. Each small window (2 s, 100 µV) shows the data on an individual electrode. The grey horizontal line in each window represents the signal detection threshold (-5 × standard deviation of noise). The overviews are shown for MEAs with **(a)** 25 nm, **(b)** 45 nm and **(c)** 65 nm thick ALD TiN electrodes, as well as a commercial control MEA
(d). The displayed traces were recorded 3 **(b)**, 7 **(a)** and 14 **(c,d)** days after plating on MEA. In **(a-c)** the third and fourth electrodes from the top in the left- and right-most columns did not have corresponding standard electrodes in grid, which causes the aberrant noise levels. In **(b)**, the high noise level in the fourth electrode from the top in the fifth column from the left was caused by a temporary poor connection between the contact pin in the headstage and the contact pad in the MEA. Only a few spikes are visible because the view shows only 2 s of data at a time and because it takes more than 3-14 days for hiPSC-derived neuronal networks to reach maximal activity.
